# Mitral valve repair and concomitant maze procedure versus catheter ablation in the treatment of atrial functional mitral regurgitation

**DOI:** 10.1186/s12872-022-02972-4

**Published:** 2022-12-12

**Authors:** Xingli Fan, Yangfeng Tang, Ye Ma, Boyao Zhang, Jie Lu, Lin Han, Yongbing Chen

**Affiliations:** 1 The Second Affiliated Hospital of Soochow University, Suzhou, 215004 Jiangsu China; 2 Department of Cardiovascular Surgery, Changhai Hospital, Naval Military Medical University, Shanghai, 200433 China; 3 Department of Cardiothoracic Surgery, The Second Affiliated Hospital of Soochow University, Suzhou, 215004 Jiangsu China

**Keywords:** Atrial functional mitral regurgitation, Atrial fibrillation, Mitral valve repair, Maze procedure, Catheter ablation

## Abstract

**Background:**

To compare mitral valve (MV) repair and concomitant maze procedure with catheter ablation in treating patients with atrial functional mitral regurgitation (AFMR).

**Methods:**

We retrospectively identified 126 patients with AFMR from January 2012 to December 2015. Of these patients, 60 patients underwent MV repair and concomitant maze procedure, and 66 patients received catheter ablation. Patients were followed up for 7.98 ± 2.01 years. The survival, readmission of heart failure (HF), persistent atrial fibrillation (AF), persistent moderate-severe mitral regurgitation (MR) and tricuspid Regurgitation (TR), and echocardiographic data were analyzed in the follow-up. Predictors of readmission of HF were analyzed.

**Results:**

There was no significant difference in baseline and echocardiographic characteristics, in-hospital mortality, and other adverse events postoperatively between two groups. The surgical group was associated with lower rates of MR > 2 + grade either at discharge (*P* = 0.0023) or in the follow-up (*P* = 0.0001). There was no significant difference in the incidence of overall survival between the two groups. The surgical group was associated with a lower rate of readmission of HF and AF in the follow-up. Univariable and multivariable analysis confirmed AF at discharge, moderate-severe MR at discharge, no MV surgery, moderate-severe TR at discharge, and LA volume as predictors of readmission of HF. Both groups experienced significant reverse cardiac remodeling.

**Conclusions:**

Our results suggest that for the treatment of AFMR with persistent or long-standing persistent AF and moderate-severe MR, MV repair and concomitant maze procedure may achieve a better outcome than catheter ablation procedure.

## Background

Mitral regurgitation (MR) can develop in individuals who have left atrium (LA) dilatation, which causes enlargement of the mitral annulus (MA) and leaflet malcoaptation [1, 2]. Atrial fibrillation (AF) is the most prevalent cause of LA advancement and annular dilatation, which leads to central regurgitation and worsens the severity of MR [3]. Atrial functional mitral regurgitation (AFMR), the disease that was reported, is frequently associated with heart failure with maintained ejection fraction (HFpEF) [3].

Previous research has shown that AFMR has a unique pathophysiology and could be treated in a variety of ways. A sinus rhythm restoration strategy contributes to the improvement of valve function in patients with AFMR [4]. Two small studies that enrolled 10 and 20 cases of AFMR, respectively, reported good short-term outcomes after mitral valve (MV) repair [5, 6]. In this study, we identified a number of patients with AFMR and compared the MV repair and concomitant maze procedure with the catheter ablation in treating this pathophysiology.

## Methods

### Patients

The retrospective study was approved by the Ethics Committee of Changhai Hospital affiliated to Naval Military Medical University (No. 20220216; February 16, 2022). All patients have been given an opt-out participant information and signed informed consent for treatment. 126 patients with AFMR who had undergone MV repair and a concurrent maze procedure (the surgical group) in the department of cardiovascular surgery and 66 patients who had undergone catheter ablation surgery (the ablation group) in the department of cardiology were eligible for inclusion between January 2012 and December 2015. All of the patients were diagnosed with chronic HF symptoms, New York Heart Association (NYHA) functional class II or higher, persistent or long-standing persistent AF, moderate to severe MR, and left ventricular ejection fraction (LVEF) ≥ 50%. Patients were excluded if they had the following: organic MR (flail leaflet, leaflet prolapsed, chordae ruptured or elongated, annular calcification, leaflet thickening, rheumatic heart disease, leaflet perforation, cleft leaflet, parachute MV, ischemic heart disease, or cardiomyopathy), LVEF < 50%, coronary artery disease (CAD), left atrial thrombus, and paroxysmal AF. The guidelines define long-standing persistent AF as continuous AF of > 12 months' duration when it is decided to adopt a rhythm control strategy [7]. Persistent AF is defined as AF continuously sustained beyond 7 days, including episodes terminated by cardioversion (drugs or electrical cardioversion) after 7 days. Clinical and demographic information for every patient was gathered retroactively.

### Surgical technique

The patients in the surgical group underwent median sternotomy. Cardiopulmonary bypass (CPB) was established by ascending aortic cannulation and bicaval venous return. The maze procedure was performed using bipolar radio frequency ablation (Atricure, Inc, Cincinnati, Ohio) with the heart arrested. The LA appendage was removed after the right Pulmonary vein (PV) were isolated, and then the left PV were ablated in a similar fashion. Each application was repeated three times. Two ablation lines were created from the right inferior PV to the left inferior PV and from the right superior PV to the left superior PV to create connecting lesions. The box lesion to the mitral annulus was created with mono-polar device with a view to reducing recurrence of AF. The right atrial maze procedures were accomplished by creating an ablation line from the superior vena cava to the inferior vena cava, an ablation line from the right atrial appendage to the atrioventricular groove, and a caudal right atrial ablation line from the atrioventricular groove to the inter atrial septum. The MV was exposed through an inter-atrial groove incision. After sizing of the intercommissural distance and anterior MV size, an annuloplasty ring (Carpentier-Edwards Physio Ring, Edwards Life Sciences, Irvine, USA; Duran Ancore Annuloplasty, Medtronic, Minneapolis, USA) was implanted. If there was a large gap between the anterior mitral leaflet and the posterior mitral leaflet owing to severe posterior leaflet tenting, posterior leaflet augmentation using autologous pericardium was performed. If moderate-severe tricuspid Regurgitation (TR) existed, the concomitant tricuspid annuloplasty (TAP) procedure was performed via DeVega technique or implanting an annuloplasty ring (BalMedic annuloplasty, BalMedic, Beijing, China).

### Catheter ablation procedure

All patients underwent pulmonary vein electrical isolation, left atrial fragmentation potential ablation, mitral annulus isthmus linear ablation, left atrium top linear ablation, and cavo-tricuspid isthmus ablation. The ablation endpoint was both persistent PV isolation and no AF with repeat incremental infusion of up to 20 ug/min of isoproterenol, as previously described [4].

### Echocardiographic analysis

All patients had a transthoracic echocardiogram (TTE) (Vivid E9, General Electric Company, Boston, USA) perioperatively and in follow-up. To assess the result of MV repair, patients in the surgical group had an intraoperative transesophageal echocardiogram (TEE). The severity of MR and TR were defined using an assessment of the color Doppler-derived jet area, the effective regurgitant orifice area using the proximal isovelocity surface area method. The morphology and dimensions of the LA and LV were also measured [8]. The LA volume, left ventricular end-diastolic dimension (LVEDD), left ventricular end-systolic dimension (LVESD), and interventricular septum thickness (IVST) were measured according to guidelines [9]. All echocardiograms were interpreted by one experienced cardiologists with level III training, and the echocardiography readers were not blinded to the patient data.

### Patient follow-up

Patients were routinely treated with anti-arrhythmic medications. Anti-arrhythmic medications were typically discontinued at 6 to 12 weeks if patients had paroxysmal AF and at 6 months if they had persistent AF, but were continued beyond this point in selected patients based on doctor and/or patient preference even in the absence of an arrhythmia event. The patients who were implanted with an annuloplasty ring were treated with warfarin for at least 3 months in the surgical group. Warfarin was definitely given to all patients who stayed in AF but was stopped 6 months later if they reverted to sinus rhythm.

All preoperative and postoperative data, including clinical, electrocardiogram, and echocardiographic findings, were obtained from the institutional database. Follow-up information was collected through a telephone interview with surviving patients or an outpatient appointment. All patients' follow-up lasted 7.98 2.01 years and was completed 100% of the time. The observed indicators included the overall mortality, the readmission for HF, the MR degree, the recurrence of AF, the cardiac remodeling values assessed by echocardiography, and the rhythm assessed by Holter electrocardiogram in the follow-up. Electrocardiogram and echocardiographic follow-up findings were obtained by querying the institutional database.

### Statistical analysis

Statistical analysis was performed with the Statistical Package for Social Sciences 19.0 (SPSS, Inc., Chicago, IL, USA). All continuous variables were expressed as mean ± standard deviation and were tested for normality. If the continuous variables conform the normal distribution, the Student t-test was used for comparison; otherwise, the Wilcoxon rank-sum test was used for comparison. Categorical variables were expressed as percentages and compared according to the Pearson chi-square test, Continuity corrected chi-square test or Fisher exact test. The univariable and multivariable analysis were estimated by the Logistic regression analysis. For all time-to-event analyses, rates were estimated by the method of Kaplan–Meier and compared by the log-rank test. A *P* < 0.05 was considered statistically significant. The power calculation made to minimal participant number was 0.9998.

## Results

The patient characteristics and preoperative echocardiographic findings are summarized in Table 1. There were no significant differences in the above indices between the two groups. Table 2 summarizes the perioperative findings. All patients in the surgical group were implanted with an annuloplasty ring; 10 (20.0%) patients underwent a posterior leaflet augmentation procedure. TAP was performed in all patients with moderate-to-severe TR. The ablation procedure was successfully completed in all patients in the ablation group. Four patients had moderate-to-severe MR in the surgical group compared with 18 patients in the ablation group (6.7% versus 27.2%, *P* = 0.0023); 1 patient had moderate-to-severe TR in the surgical group compared with 16 patients in the ablation group (1.7% versus 24.2%, *P* = 0.0006); and 8 patients had persistent AF in the surgical group compared with 12 patients in the ablation group (13.3% versus 18.2%, *P* = 0.4570) postoperatively. There was no significant difference in in-hospital mortality, surgical intervention for bleeding, significant bradycardia, or stroke/TIA between the two groups.

**Table 1:** Baseline characteristics and preoperative echocardiographic data

Variables	Surgical group (%)	Ablation group (%)	*P* value
Baseline characteristics			
Age(years)	63 ± 5.7	65 ± 5.9	0.3697
Male	38 (63.3)	40 (60.6)	0.3235
BSA(m^2^)	1.68 ± 0.16	1.66 ± 0.12	0.5247
Hypertension	16 (26.7)	18 (27.3)	0.9390
Diabetes	10 (16.7)	13 (19.7)	0.6601
COPD	4 (6.7)	5 (7.6)	1.0000
Coronary disease	9 (15.0)	10 (15.1)	0.9811
Previous MI	2 (3.3)	4 (6.1)	0.7648
Previous stroke/TIA	7 (11.7)	10 (15.1)	0.5674
Renal failure	1 (1.7)	1 (1.5)	1.0000
NYHA classifications			
I ~ II	21 (35.0)	25 (37.9)	0.7375
III	39 (65.0)	41 (62.1)	0.7375
IV	0	0	–
Duration of AF (months)	19 ± 9.6	20 ± 7.6	0.4124
Previous AF ablations	0	0	–
EuroSCORE II, %	2.5 (1.6–4.1)	2.4 (1.2–4.0)	0.5128
STS PROM score, %	3.7 (2.2–5.8)	3.5 (2.0–5.7)	0.4682
Echocardiographic characteristics			
LA volume (ml)	119 ± 49	116 ± 45	0.2927
LVEDD (mm)	49 ± 6.0	48 ± 6.8	0.3061
LVESD (mm)	37 ± 6.1	38 ± 6.4	0.2167
IVS (mm)	10 ± 1.0	10 ± 0.8	0.5871
LVEF (%)	56 ± 8.2	58 ± 8.5	0.3150
Moderate-severe MR	60 (100)	66 (100)	0.1994
Moderate-severe TR	46 (76.7)	50 (75.8)	0.9048

*BSA* body surface area, *COPD* chronic obstructive pulmonary disease, *MI* myocardial infarction, *TIA* transient ischemic attack, *NYHA* New York heart association, *AF* atrial fibrillation, *LA* left atrium, *LVEDD* left ventricular end diastolic diameter, *LVESD* left ventricular end systolic diameter, *IVS* interventricular septum, *LVEF* left ventricular ejection fraction, *MR* mitral regurgitation, *TR* tricuspid Regurgitation

**Table 2:** Operative and postoperative characteristics

Variables	Surgical group (%)	Ablation group (%)	*P* value
Operative characteristics			
CPB time (min)	108 ± 28.8	–	–
Cross-clamp time (min)	40 ± 7.5	–	–
MV annuloplasty ring	60 (100)	–	–
Posterior leaflet augmentation	12 (20.0)		
TAP	46 (76.7)		
Maze procedure	60 (100)	–	–
Catheter ablation procedure	–	66 (100)	–
Postoperative characteristics			
In-hospital mortality	0	0	**–**
Surgical intervention for bleeding	1 (1.7)	2 (3.0)	1.0000
Significant bradycardia	1 (1.7)	1 (1.5)	1.0000
Stroke/TIA	1 (1.7)	1 (1.5)	1.0000
ICU length of stay (days)	3 ± 0.8	1 ± 0.5	0.0281
Hospital length of stay (days)	12 ± 3.6	6 ± 1.8	0.0012
Acute kidney injury	2 (3.3)	0	0.2248
Wound infection	1 (1.7)	0	0.4762
Moderate-severe MR	4 (6.7)	18 (27.2)	0.0023
Moderate-severe TR	1 (1.7)	16 (24.2)	0.0006
AF	8 (13.3)	12 (18.2)	0.4570

*CPB* cardiopulmonary bypass, *MV* mitral valve, *TAP* tricuspid annuloplasty, *TIA* transient ischemic attack, *MR* mitral regurgitation, *TR* tricuspid regurgitation, *AF* atrial fibrillation

**Fig. 1:**
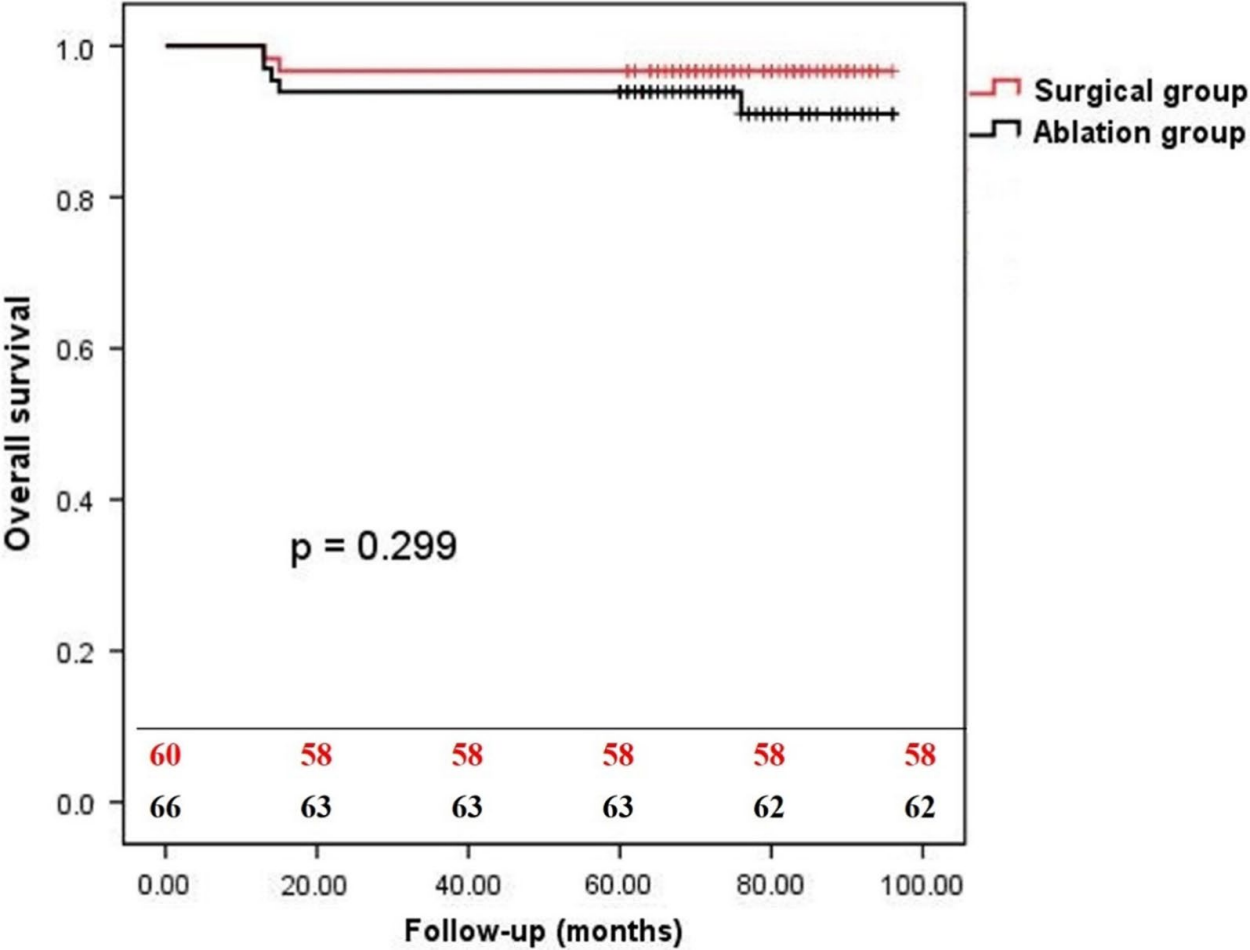
The overall survival of patients with AFMR underwent mitral valve repair and concomitant maze procedure (surgical group), or catheter ablation (ablation group). There was no significant difference between two groups (*P* = 0.2934)

**Fig. 2:**
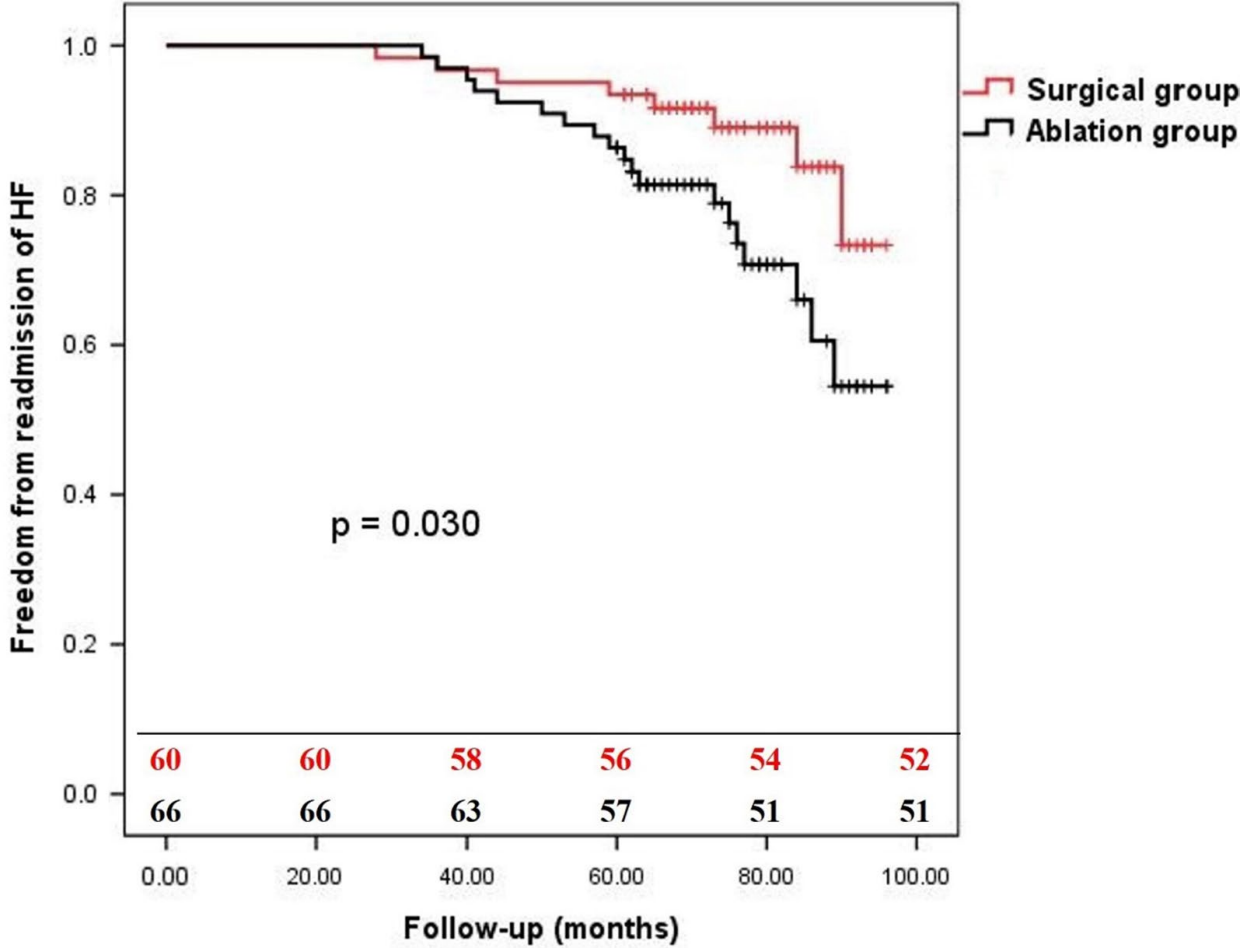
The freedom from readmission of HF of patients with AFMR in the surgical group and the ablation group. Patients in ablation group showed a significant high incidence of readmission of HF (*P* = 0.0296)

**Fig. 3:**
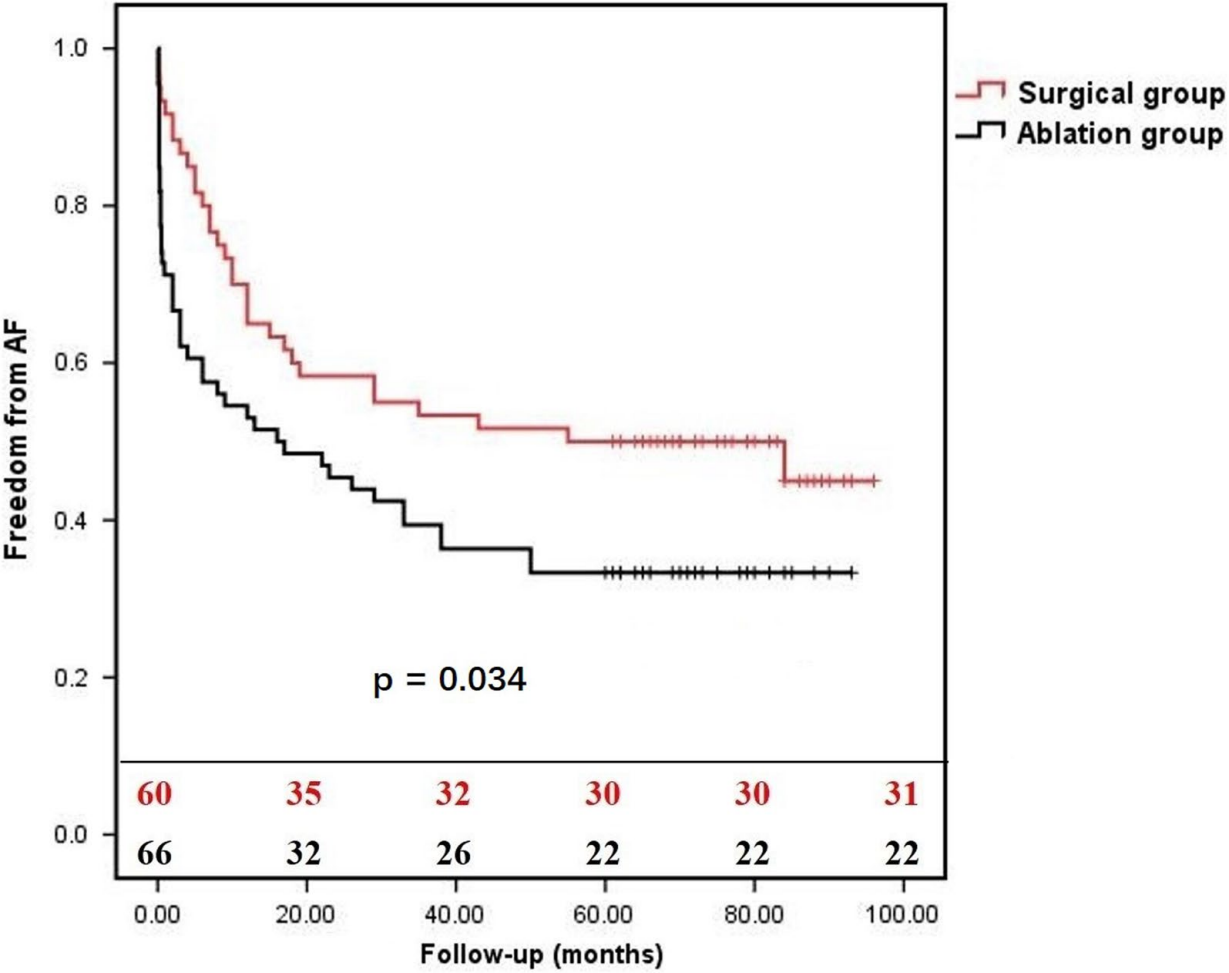
The freedom from AF of patients with AFMR in the surgical group and the ablation group. Patients in ablation group showed a significant high incidence of AF (*P* = 0.034)

**Fig. 4:**
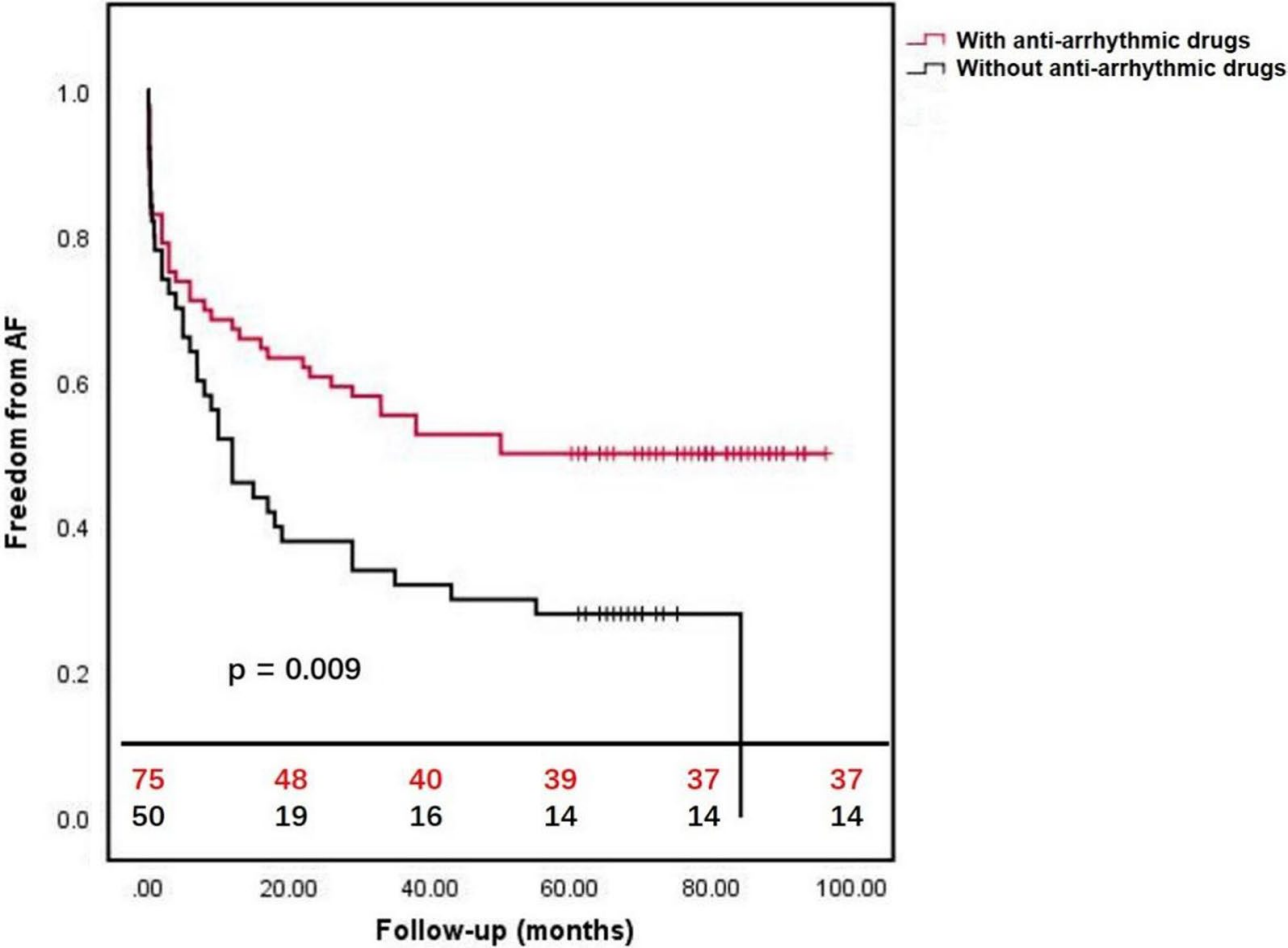
The freedom from AF of patients with AFMR with and without anti-arrhythmic drugs after surgery or ablation

In the follow-up, moderate-severe MR occurred in 10 patients in the surgical group compared with in 35 patients in the ablation group (16.7% versus 53.0%, *P* = 0.0001), and moderate-severe TR occurred in 6 patients in the surgical group compared with in 20 patients in the ablation group (10.0% versus 30.3%, *P* = 0.0037). The overall survival rates at 1-year and 5-year follow-up were 100% and 96.7% for the surgical group versus 100% and 93.9% for the ablation group (*P* = 0.299) (Fig. 1). The freedom from readmission of HF at 1-year and 5-year follow-up were 100% and 93.4% for the surgical group versus 100% and 86.4% for the ablation group (*P* = 0.030) (Fig. 2). The freedom from AF at 1-year and 5-year follow-up were 65.0% and 50.0% for the surgical group versus 53.0% and 33.3% for the ablation group (*P* = 0.034) (Fig. 3). The freedom from AF at 1-year and 5-year follow-up were 67.1% and 50.0% for the patients with anti-arrhythmic drugs versus 46.0% and 28.0% for the patients without anti-arrhythmic drugs (*P* = 0.009) (Fig. 4).

**Fig. 5:**
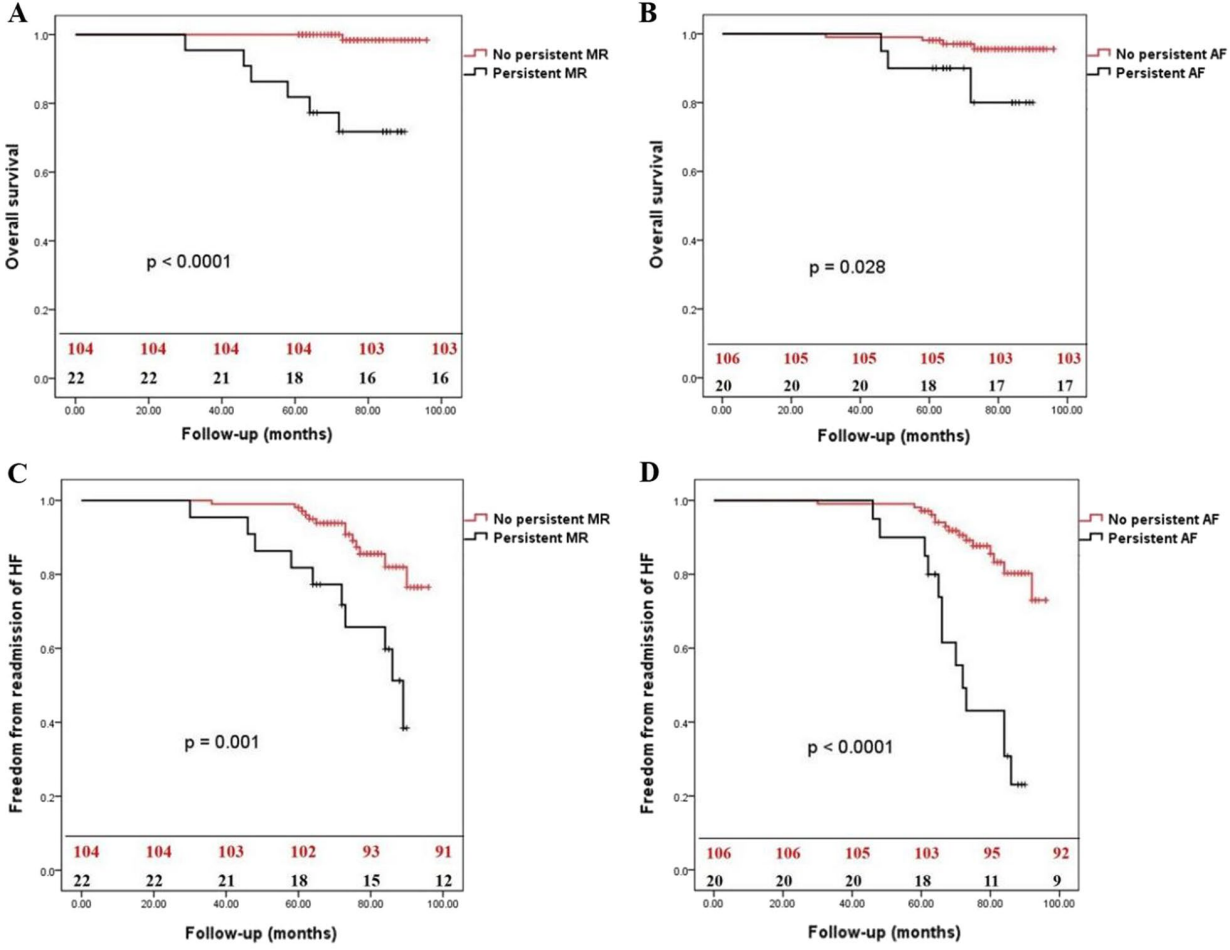
The overall survival and freedom from readmission of HF of patients with or without persistent MR and persistent AF at discharge. **A** represents the overall survival of patients with or without persistent MR at discharge (*P* < 0.0001), **B** represents the overall survival of patients with or without persistent AF (*P* = 0.028), **C** represents the freedom from readmission of HF of patients with or without persistent MR at discharge (*P* = 0.001), **D** represents the freedom from readmission of HF of patients with or without persistent AF at discharge (*P* < 0.0001)

The univariable and multivariable analysis confirmed that AF at discharge, moderate-severe MR at discharge, no MV surgery, moderate-severe TR at discharge, and LA volume as predictors of readmission of HF (Table 3). Patients with persistent moderate-severe MR and persistent AF early after surgery had poorer survival (Fig. 5A and B) and high incidence of readmission of HF (Fig. 5C and D).

**Table 3:** Univariable and multivariable analysis of readmission of HF

Variables	Univariable		Multivariable	
	OR (95% CI)	*P* value	OR (95% CI)	*P* value
Age	1.089 (0.998–1.099)	0.3792		
Male	2.356 (0.425–11.564)	0.2493		
BSA	2.674 (0.504–11.875)	0.2134		
Hypertension	2.128 (0.346–6.693)	0.4246		
Diabetes	1.136 (0.595–4.184)	0.3462		
COPD	1.245 (0.643–5.743)	0.2522		
Coronary disease	2.875 (0.612–12.363)	0.2953		
Renal failure	3.644 (0.960–13.754)	0.0814		
EuroSCORE II, %	1.321 (0.692–4.643)	0.4257		
STS PROM score, %	1.295 (0.674–4.256)	0.4369		
Duration of AF	2.199 (0.358–6.709)	0.4987		
LA volume	2.985 (1.193–6.938)	0.0421	2.019 (1.008–6.085)	0.0329
LVEDD	2.194 (0.351–6.632)	0.6839		
LVESD	2.283 (0.401–6.928)	0.7024		
IVS	1.758 (0.801–5.081)	0.5932		
LVEF	3.674 (0.977–13.927)	0.0838		
No MV surgery	5.374 (1.664–15.129)	0.0009	5.062 (1.562–14.671)	0.0011
AF at discharge	3.392 (1.172–9.045)	0.0098	3.151 (1.021–8.283)	0.0124
Moderate-severe MR at discharge	6.013 (2.094–17.125)	0.0003	5.823 (1.968–16.374)	0.0009
Moderate-severe TR at discharge	4.359 (1.903–15.937)	0.0008	4.023 (1.828–15.195)	0.0014

*OR* odds ratio, *CI* confidence interval, *BSA* body surface area, *COPD* chronic obstructive pulmonary disease, *AF* atrial fibrillation, *LA* left atrium, *LVEDD* left ventricular end diastolic diameter, *LVESD* left ventricular end systolic diameter, *IVS* interventricular septum, *LVEF* left ventricular ejection fraction, *MV* mitral valve, *MR* mitral regurgitation, *TR* tricuspid Regurgitation

Both groups experienced significant reverse cardiac remodeling in the follow-up, but this effect was more evident in the surgical group (Table 4). The mean reduction of LA volume is 39 ± 9.3 ml (*P* < 0.0001) in the surgical group and 21 ± 6.8 ml (*P* = 0.0366). There was no difference in the variation of LVEDD, LVESD, IVS, and LVEF in the two groups.

**Table 4:** Reverse of cardiac remodeling in the follow-up

Variables	Surgical group			Ablation group		
	MD	95% CI	*P* value	MD	95% CI	*P* value
LA volume (ml)	− 39	16.77–59.23	< 0.0001	− 21	− 0.23–42.23	0.0366
LVEDD (mm)	− 1.7	− 0.11–0.45	0.4805	− 1.0	− 0.16–0.39	0.5023
LVESD (mm)	− 2.2	− 0.05–0.49	0.2018	− 1.7	− 0.11–0.44	0.4796
IVST (mm)	0.02	− 0.02–0.21	0.7849	0.01	− 0.03–0.20	0.7919
LVEF (%)	3.0	− 0.77–6.77	0.1424	1.6	− 0.84–6.03	0.1944

*MD* mean displacement, *CI* confidence interval, *LA* left atrium, *LVEDD* left ventricular end diastolic diameter, *LVESD* left ventricular end systolic diameter, *IVST* interventricular septum thickness, *LVEF* left ventricular ejection fraction

## Discussion

In this study, we compared mitral valve repair and concomitant maze procedure with catheter ablation procedure for the treatment of patients with AFMR. According to the findings of this study, the patients who underwent surgical procedure had more improved MR and TR at discharge, a lower incidence of HF and AF in the follow-up, and lower recurrence rates of moderate-severe MR and TR in the follow-up. Furthermore, we identified that AF at discharge, moderate-severe MR at discharge, no MV surgery, moderate-severe TR at discharge, and LA volume were the predictors of readmission of HF.

AFMR was mentioned for the first time by Gerts et al. in 2011 [4]. This type of MR is characterized by preserved LV systolic function and AF, which induces a LA dilation, thus leading to annular dilation and malcoaptation of the leaflets. Kim et al. and Kagiyama et al. found that the ratio of total leaflet area to MA area was significantly smaller in patients with AF and MR than in those without MR and a normal valve [10, 11]. These findings show that insufficient leaflet adaptation is a risk factor for MR in patients with AFMR. Furthermore, AF impaired the annular dynamics and "saddle" structure that affected the involution of leaflets and may contribute to the MR [12].

The optimal treatment strategy for AFMR is still limited due to the special pathological mechanisms. Successful ablation of AF may be beneficial in reducing MR. Gertz et al. found that restoration of sinus rhythm significantly improved MR and induced reverse LA remodeling at 1-year in 53 patients with AFMR [4].Takahashi et al. conducted another study that enrolled 45 patients with AFMR [13]. This research indicated that mitral repair reduced MR and relieved symptoms, but was not sufficient to prevent cardiovascular events in patients with a large LA. The presence of a large LA was a significant predictor of postoperative valve-related mortality, heart failure readmission, and postoperative cardiovascular events. In contrast to Gertz’s study, the LA of the included population was larger in the above-mentioned research. This indicated that the atrial remodeling of patients in the surgical management studies involved was more serious and that the disease had developed to a more advanced stage. In our study, the populations presented with a large LA, and the surgical management significantly reduced the readmission of HF and induced reverse LA remodeling compared to catheter ablation. Furthermore, long-standing persistent AF is often associated with severely dilated LA (> 65 mm) [14] and MA, meaning isolated catheter ablation might be insufficient. These observations may support the hypothesis that surgical management is more suitable for advanced stages of AFMR than catheter ablation, which might be defined by severely dilated LA and MA and significant LA remodeling.

The implementation of the maze procedure in this investigation was higher compared to those of the above-mentioned studies, which may have contributed to the postoperative maintenance of sinus rhythm. Chen et al. compared mitral repair with concomitant surgical ablation to isolated mitral repair in treating patients with AFMR [15]. The results indicated that patients may benefit from a concurrent surgical ablation operation in terms of recurrent MR. It can thus be suggested that, for the treatment of patients with AFMR, additional surgical ablation on the basis of mitral repair may be a significantly reasonable strategy. However, patients with a giant LA and long-standing persistent AF are usually refractory to the maze procedure, and the restoration of sinus rhythm is difficult. Further study should elucidate the reasonable indication of the maze procedure in the treatment of patients with AFMR.

This study had some limitations. This was a single-center retrospective research with a small sample size which may lead to bias. Additionally, this study did not show the size of annuloplasty ring which may be associated with the efficiency of surgery. Finally, further research is necessary to evaluate the treatment strategies for patients with AFMR in different periods.

## Conclusions

Patients with AFMR who undergo surgical procedure had more improved MR and TR at discharge, lower incidence of HF and AF in the follow-up, and lower recurrence rates of moderate-severe MR and TR in the follow-up than those who undergo catheter ablation procedure. For the treatment of AFMR with persistent or long-standing persistent AF and moderate-severe MR, MV repair and concomitant maze procedure may achieve a better outcome than catheter ablation procedure.

## Data Availability

The datasets used and/or analysed during the current study are available from the corresponding author on reasonable request.

## Abbreviations

MR: Mitral regurgitation
LA: Left atrium
MA: Mitral annulus
MV: Mitral valve
AFMR: Atrial functional mitral regurgitation
HF: Heart failure
AF: Atrial fibrillation
HFpEF: Heart failure with maintained ejection fraction
MV: Mitral valve
NYHA: New York Heart Association
LVEF: Left ventricular ejection fraction
CAD: Coronary artery disease
CPB: Cardiopulmonary bypass
PV: Pulmonary vein
TR: Tricuspid Regurgitation
TAP: Tricuspid annuloplasty
TTE: Transthoracic echocardiogram
TEE: Transesophageal echocardiogram
LVEDD: Left ventricular end-diastolic dimension
LVESD: Left ventricular end-systolic dimension
IVST: Interventricular septum thickness
OR: Odds ratio
CI: Confidence interval
BSA: Body surface area
COPD: Chronic obstructive pulmonary disease

## References

[1] Kihara T, Gillinov AM, Takasaki K, Fukuda S, Song JM, Shiota M (2009). Mitral regurgitation associated with mitral annular dilation in patients with lone atrial fibrillation: an echocardiographic study. Echocardiography (Mount Kisco, NY).

[2] Kilic A, Schwartzman DS, Subramaniam K, Zenati MA (2010). Severe functional mitral regurgitation arising from isolated annular dilatation. Ann Thorac Surg.

[3] Ennezat PV, Maréchaux S, Pibarot P, Le Jemtel TH (2013). Secondary mitral regurgitation in heart failure with reduced or preserved left ventricular ejection fraction. Cardiology.

[4] Gertz ZM, Raina A, Saghy L, Zado ES, Callans DJ, Marchlinski FE (2011). Evidence of atrial functional mitral regurgitation due to atrial fibrillation: reversal with arrhythmia control. J Am Coll Cardiol.

[5] Takahashi Y, Abe Y, Sasaki Y, Bito Y, Morisaki A, Nishimura S (2015). Mitral valve repair for atrial functional mitral regurgitation in patients with chronic atrial fibrillation. Interact Cardiovasc Thorac Surg.

[6] Vohra HA, Whistance RN, Magan A, Sadeque SA, Livesey SA (2012). Mitral valve repair for severe mitral regurgitation secondary to lone atrial fibrillation. Eur J Cardio-Thoracic Surg Off J Eur Assoc Cardio-thoracic Surg.

[7] Hindricks G, Potpara T, Dagres N, Arbelo E, Bax JJ, Blomström-Lundqvist C (2021). 2020 ESC Guidelines for the diagnosis and management of atrial fibrillation developed in collaboration with the European Association for Cardio-Thoracic Surgery (EACTS): The Task Force for the diagnosis and management of atrial fibrillation of the European Society of Cardiology (ESC) Developed with the special contribution of the European Heart Rhythm Association (EHRA) of the ESC. Eur Heart J.

[8] Zoghbi WA, Adams D, Bonow RO, Enriquez-Sarano M, Foster E, Grayburn PA (2017). Recommendations for noninvasive evaluation of native valvular regurgitation: a report from the American Society of Echocardiography Developed in collaboration with the society for cardiovascular magnetic resonance. J Am Soc Echocardiogr Off Publ Am Soc Echocardiogr.

[9] Lang RM, Badano LP, Mor-Avi V, Afilalo J, Armstrong A, Ernande L (2015). Recommendations for cardiac chamber quantification by echocardiography in adults: an update from the American Society of Echocardiography and the European Association of Cardiovascular Imaging. J Am Soc Echocardiogr Off Publ Am Soc Echocardiogr.

[10] Kim DH, Heo R, Handschumacher MD, Lee S, Choi YS, Kim KR (2019). Mitral valve adaptation to isolated annular dilation: insights into the mechanism of atrial functional mitral regurgitation. JACC Cardiovasc Imaging.

[11] Kagiyama N, Hayashida A, Toki M, Fukuda S, Ohara M, Hirohata A (2017). Insufficient leaflet remodeling in patients with atrial fibrillation: association with the severity of mitral regurgitation. Circ Cardiovasc Imaging.

[12] Levack MM, Jassar AS, Shang EK, Vergnat M, Woo YJ, Acker MA (2012). Three-dimensional echocardiographic analysis of mitral annular dynamics: implication for annuloplasty selection. Circulation.

[13] Takahashi Y, Abe Y, Takashi M, Fujii H, Morisaki A, Nishimura S (2020). Mid-term results of valve repairs for atrial functional mitral and tricuspid regurgitations. Gen Thorac Cardiovasc Surg.

[14] Apostolakis E, Shuhaiber JH (2008). The surgical management of giant left atrium. Eur J Cardio-Thoracic Surg Off J Eur Assoc Cardio-Thoracic Surg.

[15] Chen J, Wang Y, Lv M, Yang Z, Zhu S, Wei L (2020). Mitral valve repair and surgical ablation for atrial functional mitral regurgitation. Ann Transl Med.

